# Periodicity: A Characteristic of Heart Rate Variability Modified by the Type of Mechanical Ventilation After Acute Lung Injury

**DOI:** 10.3389/fphys.2018.00772

**Published:** 2018-06-19

**Authors:** Anurak Thungtong, Matthew F. Knoch, Frank J. Jacono, Thomas E. Dick, Kenneth A. Loparo

**Affiliations:** ^1^School of Engineering and Resources, Walailak University, Nakhon Si Thammarat, Thailand; ^2^Department of Electrical Engineering and Computer Science, Case Western Reserve University, Cleveland, OH, United States; ^3^Division of Pulmonary, Critical Care & Sleep Medicine, Department of Medicine, University Hospitals Cleveland Medical Center (UHCMC), Cleveland, OH, United States; ^4^Louis Stokes Cleveland VA Medical Center, Cleveland, OH, United States; ^5^Department of Neurosciences, Case Western Reserve University, Cleveland, OH, United States

**Keywords:** periodicity, HRV, continuous mechanical ventilation, biologically variable ventilation, periodogram

## Abstract

We present a novel approach to quantify heart rate variability (HRV) and the results of applying this approach to synthetic and original data sets. Our approach evaluates the periodicity of heart rate by calculating the transform of Relative Shannon Entropy, the maximum value of the RR interval periodogram, and the maximum, mean values, and sample entropy of the autocorrelation function. Synthetic data were generated using a Van der Pol oscillator; and the original data were electrocardiogram (ECG) recordings from anesthetized rats after acute lung injury while on biologically variable (BVV) or continuous mechanical ventilation (CMV). Analysis of the synthetic data revealed that our measures were correlated highly to the bandwidth of the oscillator and assessed periodicity. Then, applying these analytical tools to the ECGs determined that the heart rate (HR) of BVV group had less periodicity and higher variability than the HR of the CMV group. Quantifying periodicity effectively identified a readily apparent difference in HRV during BVV and CMV that was not identified by power spectral density measures during BVV and CMV. Cardiorespiratory coupling is the probable mechanism for HRV increasing during BVV and becoming periodic during CMV. Thus, the absence or presence of periodicity in ventilation determined HRV, and this mechanism is distinctly different from the cardiorespiratory uncoupling that accounts for the loss of HRV during sepsis.

## Introduction

Heart rate variability (HRV) refers to the variation in beat-to-beat intervals (RR interval) and is measured from the electrocardiogram (ECG). Multiple analytic tools have been applied to quantify HRV in both the frequency and time domains. Generally, power spectral analysis is applied to quantify HRV in the frequency domain and changes in relative power in defined bands may reflect changes in: (1) the balance of the autonomic nervous system (Stein et al., 1994; Goldberger et al., 2001; Sztajzel, 2004), (2) clinical status of disease states such as cardiovascular disease (Hillebrand et al., 2013; Schuster et al., 2016) and diabetes (Karmakar et al., 2015; Wulsin et al., 2015; Ziegler et al., 2015), and (3) stress (Shah et al., 2015; Williams et al., 2015). These studies revealed that disease can reduce HRV. Analysis of HRV in the time domain complements its analysis in the frequency domain. Mean HR plus and minus standard deviation is a common but rarely referred to as a time domain analysis of the variation of RR interval even though the coefficient of variation can be derived by dividing the standard deviation by the mean. Poincaré circle-return maps plot the RR interval of n+t against the RR interval of n. This displays the distribution of RR intervals as they relate to the prior RR interval in plots where t = 1; or a previous RR interval, where t > 1. The distribution of points in these plots often can be visualized by an ellipse whose long axis lies on the line of identity. The short-axis is interpreted as an index of short-term HRV and; similarly the long axis, long-term HRV (Brennan et al., 2001; Fishman et al., 2012). During the last 20 years, various nonlinear analyses have been applied to assess HRV such as: detrended fluctuation analysis, to detect long-range correlation (Penzel et al., 2003); sample entropy to measure the irregularity of a RR interval time series (Richman and Moorman, 2000); and temporal pattern variability analysis, to quantify the nonlinear and temporal information encoded in the Poincaré plot (Fishman et al., 2012). The details of these tools was summarized in the literature (Shaffer and Ginsberg, 2017).

Biologically variable ventilation (BVV) refers to a method of mechanical ventilation that imposes variability in cycle duration and tidal volume. It is biologic in that the ventilator's pattern is designed to reproduce normal breathing variations, and is often based on a recorded spontaneous breathing pattern. In contrast, continuous mechanical ventilation (CMV), refers to mechanical ventilation that imposes a nonvarying rate and tidal volume (Naik et al., 2015). As compared to CMV, BVV improves blood oxygenation and respiratory system compliance in a porcine model of oleic acid lung injury (Lefevre et al., 1996). This opened the field of critical care to the possibility that applied variability in ventilation patterns may benefit patients. Indeed, Suki et al. modeled the respiratory system *in silico* and proposed that variability improved gas exchange due to nonlinearity in lung compliance (Suki et al., 1998). Following these seminal studies, a wide range of studies report similar findings including: preclinical studies that ventilated animal models of: (1) acute lung injury/acute respiratory distress syndrome (ALI/ARDS) (Arold et al., 2002, 2003; Boker et al., 2002; Funk et al., 2004; Graham et al., 2005; Bellardine et al., 2006; Thammanomai et al., 2008; Spieth et al., 2009b) a notable exception reported no benefit of BVV (Nam et al., 2000), (2) prolonged anesthesia (Mutch et al., 2000), (3) single lung ventilation (McMullen et al., 2006), (4) severe bronchospasm (Mutch et al., 2007), and (5) others (Spieth et al., 2009a, 2012; Beda et al., 2010; Graham et al., 2011a,b; Berry et al., 2012). Further, preliminary clinical studies support use of BVV in critically ill patients with ALI (Boker et al., 2002; Kowalski et al., 2013; Spieth et al., 2014).

We have a longstanding interest in autonomic and respiratory control, in particular whether changes cardio-respiratory coupling can persist in the absence of the forces driving that coupling. For example we reported that a single 10-min period of volitional slow, deep-breathing in naïve subjects greatly enhanced cardiorespiratory coupling during that 10-min period but also affected HRV for ~10-min after returning to an eupneic pattern (Dick et al., 2014). Initially we wanted to test whether beneficiary effects on lung compliance of BVV persisted during spontaneous breathing after mechanical ventilation ceased. During these experiments, the rats received the same minute ventilation whether they received BVV or CMV and hypoxemia was prevented. We reported an approximate 15% improvement in lung compliance in BVV compared to CMV groups (Knoch et al., 2011). Thus the effect of BBV on lung function persisted in recovery. While performing these experiments, we observed an obvious effect of the mode of ventilation on HRV. However, application of the described analytical methods could not distinguish the structural differences in HRV between BVV and CMV. This manuscript describes an analytical tool we developed to measure the impact of BVV vs. CMV on HRV in adult rats that sustained lung injury and whether the differences in cardio-ventilator coupling were reflected in cardio-respiratory coupling during the recovery period.

## Materials and methods

### Recording of biologic data

We recorded, analyzed and compared periodicity in HRV in two groups of rats both of which sustained lung injury. One group was ventilated with CMV; the other, with BVV. Our protocols were approved by the Institutional Animal Care and Use Committee at Case Western Reserve University. Briefly, we anesthetized adult male rats (Sprague-Dawley/Charles River; 150–250 g; *n* = 15) with isoflurane to induce anesthesia followed by ketamine to maintain a surgical plane of anesthesia. We placed: (1) electrode leads in the diaphragm to record its electromyogram (DiaEMG) and under the skin to record the electrocardiogram (ECG) (2) a tube in the trachea to ventilate the rat and to record endotracheal pressure (PET) and (3) a MouseOx around the shaved skin (Starr Life Sciences, Oakmont, PA) to record oxygen saturation transcutaneously. The MouseOx provided a continuous recording of oxygen saturation which was maintained at 95 ± 2% throughout the experiment by adjusting the fraction of inhaled oxygen. We recorded a 60 min baseline period during which rats breathed spontaneously. Then we injured the rodents' lungs by delivering 0.1 N HCl (0.2 ml/kg) directly to the lungs via the endotracheal tube. Immediately after lung injury, we initiated either BVV (*n* = 7) or CMV (*n* = 8). Whether it was BVV or CMV, we ventilated the rats at the same average tidal volume (VT) of 7 ml/kg and the same average minute ventilation of 55 ml/kg (VR × VT). The BVV pattern was based on a plethysmograph recording of a spontaneously breathing, healthy, unanesthetized rat and was scaled to the minute ventilation of a spontaneously breathing anesthetized rat; looped every 30 min. Mechanical ventilation lasted for 4 h for both BVV and CMV groups. Spontaneous breaths occurred during ventilation and were evident in the DiaEMG and as transient decrease in PET. When the rats were removed from the ventilator, they breathed spontaneously for another hour. At the end of the recording, the rats received an overdose of ketamine and tissue samples were harvested to assess lung injury. Bronchoalveolar lavage fluid (BALF) reflected a benefit of BVV; the BALF contained fewer cells following BVV than CMV (254 ± 100 × 10^3^ cells vs. 409 ± 175 × 10^3^ cells, *P* < 0.05).

### HRV analysis of biologic data

To test our hypothesis that HRV was greater during BVV than during CMV treatment, we examined HRV using analyses in the time and frequency domains as well as nonlinear tools to assess complexity of HRV. As a first step to all these analyses, we removed artifacts and baseline drift in ECG signal and detected the R-wave peaks. In this data set, the rats were anesthetized and the ECG signal strong so artifacts were rare and not differentially distributed to a group. We calculated the HRV measures using 2.5-min epochs. For the time-domain HRV measures, we calculated the standard deviation of the RR interval time series (SDNN), the square root of the mean squared differences of the successive RR intervals (RMSSD), and the HRV triangular index that is defined by the total number of RR intervals divided by number of RR intervals in the modal bin. For the frequency-domain measures of HRV, we applied the Lomb-Scargle method to compute the periodogram (power spectral density) of the (non-uniformly sampled) RR interval time series (Rybicki and Press, 1995). We computed relative power in the low-frequency range (LF, defined as the power in the 0.1–1.0 Hz frequency range divided by the total power), relative power in the high frequency range (HF, defined as the power in the 1.0–3.5 Hz frequency range divided by the total power), and the ratio of relative powers in the low- to high- frequency ranges (LHR). These frequency bands were suggested for HRV parameters in rodents (Rowan et al., 2007). Finally, we performed a few basic nonlinear measures that included: standard deviations from the Poincaré plot (SD1, SD2, and SDRatio), short-term and long-term fluctuations from detrended fluctuation analysis (DFA α_1_, α_2_ computed within range of 16 and 64 beats, respectively), and sample entropy (SampEn) using *m* = 2 and *r* = 0.2 SDNN.

As we describe in the Results section, this analytical approach did not identify differences in HRV between the two groups, especially using the frequency domain analysis, even though a dramatic difference was apparent in the periodogram of RR interval. Thus, we developed a novel approach to measure the periodicity of the RR interval time series, a distinct measure of HRV. This analytical approach is based on differences between in the frequency spectra of periodic and aperiodic signals. Basically, the spectrum of a periodic signal is narrow and has its peak at fundamental and harmonically related frequencies; whereas, aperiodic signals have a continuous and broad spectrum. Also, this difference in the spectra should be reflected in the autocorrelation plots; the autocorrelation plot of a periodic signal is also periodic with the same period. Our proposed methods measure the differences in power spectral density and autocorrelation (summarized in sections Power Spectral Density Approach and Autocorrelation Approach).

### Power spectral density approach

This approach relies on the fact that the power spectrum of periodic and non-periodic time series are different, so we modified the spectral analysis approach to detect these differences. First, we excluded the very low frequency components (<0.3 Hz). Second, we normalized the magnitudes of the power spectral density between 0.3 and 2 Hz by dividing the magnitude at each frequency component by the total power in the signal. Then, the normalized values were plotted against frequency to display the relative power spectrum of the RR-interval time series. We quantified the relative power spectra using two methods. First, we detected the peak of relative power (maxPER, maximum of the periodogram). This measurement takes advantage of the concentration of relative power in a narrow frequency band with a high peak in periodic signal versus the dispersion of relative power over a wide frequency band with low peak values in a non-periodic signal. Conceptually, the advantages for this method are it is simple and computational efficient; the disadvantage is that the result depends on the relative power at a single frequency, which could be sensitive to influences unrelated to our experiment. Second, we evaluated the entire range of the relative power spectrum and used the transformed Relative Shannon Entropy (tRSE) to determine if the distribution of relative power of the RR-interval time series over the frequency range is uniform (Zlotnik, 2006). To compute tRSE, the frequency range was divided into 20 bins, and for each bin *i* the sum of the relative power *P(i)* was computed and the tRSE was defined as:

(1) $$\begin{array}{r} \text{tRSE }=\;1+\frac{\overset{M}{\underset{i=1}{\sum }}P\left(i\right)\ln P\left(i\right)}{\ln\;M}. \end{array}$$

If the relative power *P* is similar to the uniform distribution over the frequency range, then *P*(*i*) ≈ 1/*M*; *i* = 1,…, *M* and $tRSE\;=\;1+\frac{\ln1/M}{\ln\;M}\approx 0$. In contrast, if *P* is concentrated in a narrow frequency band centered at bin *i*, then *P*(*i*) ≈ 1 for that band and ≈ 0 at bands above or below the *i*^*th*^ band. In summary, an increasing tRSE approaching 1 indicates a narrow band signal, approximately periodic, signal. Of course, this method was more computationally intensive.

### Autocorrelation approach

In this approach, periodicity was quantified by measuring the regularity of the autocorrelation function. The autocorrelation function measures self-similarity of a time series at increasing time-delays. Given the quasi-stationarity of a RR-interval time series in an anesthetized rat, the autocorrelation function of this time series is approximately periodic. If the RR-interval varies over a large frequency range, then the autocorrelation function decreases as the time-delay increased. Before computing the autocorrelation function, the RR interval time series was detrended by dividing the time series into multiple linear segments using a change-point detection algorithm such as given in Spieth et al. (2014). Then, the time-series data in each segment was estimated using a linear least squares fit to the data. Finally, the linear trend was subtracted from each segment and the autocorrelation function was computed as:

(2) $$\begin{array}{r} R_{xx}\left(d\right)=\frac{1}{N-d}\overset{N-d}{\underset{n=1}{\sum }}{\hat{x}}_{n}{\hat{x}}_{n+d}, \end{array}$$

where x denotes the RR-interval time series, d was the delay that represents the number of heart beats rather than time because the RR-intervals are not constant, $\hat{x}=\left(x-\mu_{x}\right)/\sigma_{x}$, where μ_*x*_ and σ_*x*_ denote the mean and standard deviation of *x*, with *R*_*xx*_(*d*) ϵ [−1 1].

In this study, the degree of correlation was measured by calculating the maximum (maxACF) and mean (meanACF) of the absolute values of autocorrelation function |*R*_*xx*_(*d*)| for *d* = 25–50. The values of maxACF and meanACF are approximates 0 if the signal is random, and maxACF is approximates 1 if the signal is periodic. In addition, we measured the regularity/predictability of the autocorrelation function by computing the sample entropy (SE.ACF). If SE.ACF is close to 0, then the signal is regular/periodic, i.e., the cycle length is regular and predictable; if SE.ACF is >0 then the cycle length varies and can be complex. In our analysis, we used the false nearest neighbor technique to determine the pattern length *m* and used the first minimum of the Mutual Information (MI) of the data to select the delay τ (Fraser and Swinney, 1986; Hegger and Kantz, 1999) and set the tolerance *r* = 0.4 SD where SD was the standard deviation of *R*_*xx*_(*d*).

### Testing the analytical approaches with synthetic data

**Figure 1:**
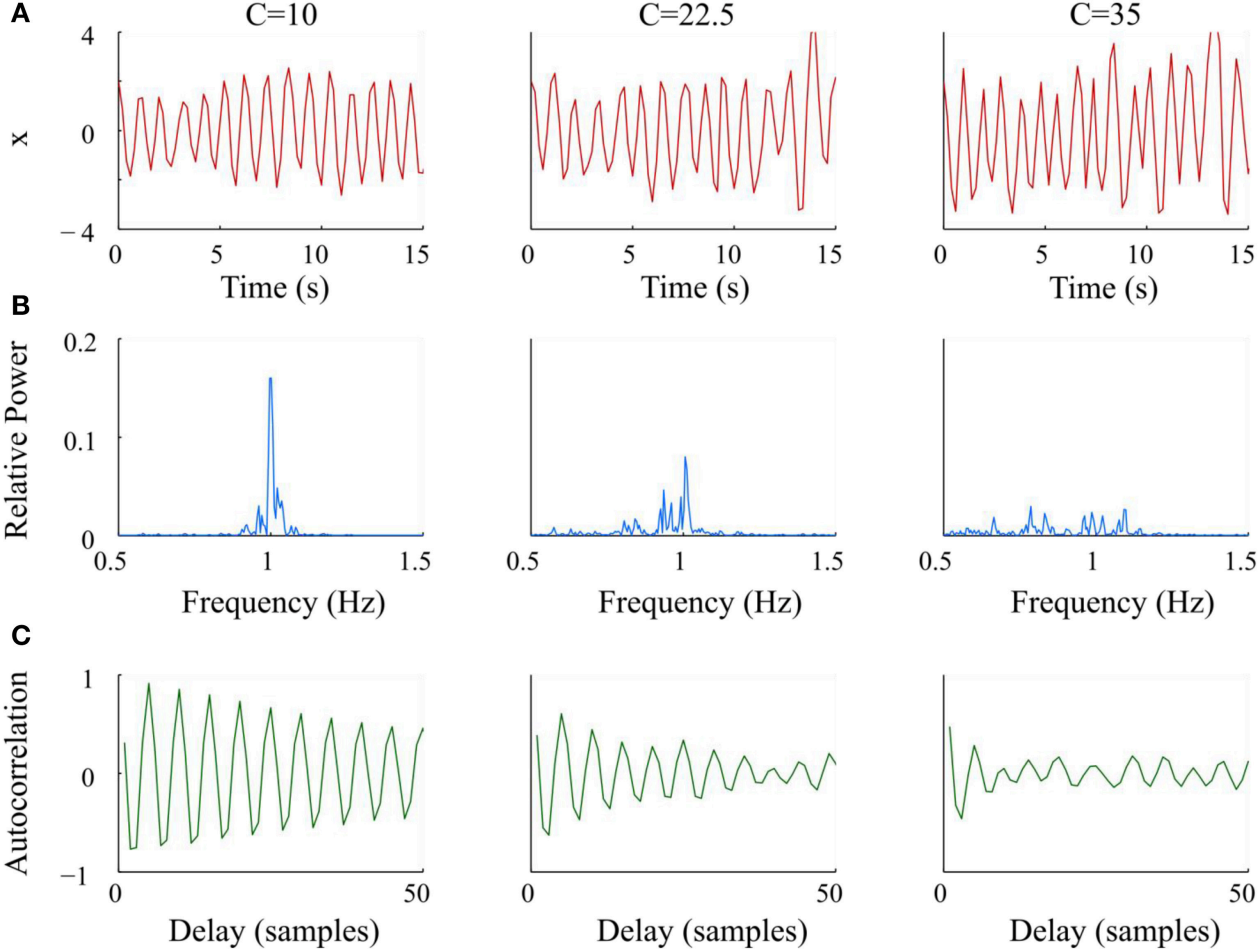
Graphs and analysis of representative synthetic data sets with increasing noise (*C*) from left to right. **(A)** Simulated Van der Pol oscillator waveform at different values of *C*. **(B)** Periodograms had wider bandwidths and lower maxima of relative power as *C* increased. **(C)** Autocorrelation functions decayed faster and lost periodicity as *C* increased.

The analytical approaches were tested on synthetic data to test their ability to quantify variability in periodic signals before they were applied to our biologic data. We replicated our RR-interval time series by simulating an oscillator with variability in a fixed bandwidth. We used the Van der Pol oscillator

(3) $$\begin{array}{r} \frac{d^{2}x}{dt^{2}}=\left(1-x^{2}\right)\frac{dx}{dt}-\left(\omega +Cz\left(t\right)\right)x\left(t\right), \end{array}$$

where ω = 40 is the parameter that controls the frequency of the oscillator. The variable *C* modulated the amplitude of the Gaussian noise *z*(*t*) ~ *N*(0, 1), which was applied directly to ω to modify the bandwidth of the oscillator. We numerically solved Equation (3) using the 4th order Runge-Kutta method with time span of 0–99.8 s and a step size of 0.2 s. Data sets were generated for 21 values of *C*, from 0 to 40 with a step size of 2.5. Figure 1 shows representative examples of the synthetic data *x*, the corresponding relative power spectra, and autocorrelation functions at 3 levels of *C*. As *C* increased, the bandwidth of the generated signal became wider; and the autocorrelation function decayed faster.

To quantitate the apparent qualitative differences in Figure 1, we generated 20 data sets for each value of *C*_*i*_, *i* = 1, …, 21 for *x*_(*i, k*)_, *k* = 1, …, 20 using different realizations of the Gaussian random noise z(t). For each of these data sets *x*_(*i, k*)_, we measured variability using our algorithms.

We compared our proposed variability measures according to their ability to detect varying bandwidths. We computed the degree of monotonicity using the algorithm modified from (Kreuz et al., 2007) as follows. Let *S* ∈ *R*^21 × 20^ of which each element *S*_(*i, k*)_ is the result of a variability measure of a data set *x*_(*i, k*)_. If *S* depends monotonically on the variable *C*, we would expect *S*_*i*_ < *S*_*k*_ for all *C*_*i*_ < *C*_*j*_, where *S*_*i*_ is the mean value of the vector *S*_*i*_ (here, *S*_*i*_ is row number *i* of matrix *S*). To determine whether *S*_*i*_ < *S*_*k*_, we used the rank sum test with significance level alpha = 0.05. If the null hypothesis *S*_*i*_ < *S*_*k*_ cannot be rejected, then *h*_(*i, j*)_ is 0, if the null hypothesis is rejected with *S*_*i*_ < *S*_*k*_, then *h*_(*i, j*)_ is−1. Thus, the degree of monotonicity is defined as:

(4) $$\begin{array}{r} M=|\frac{2}{r\left(r-1\right)}\overset{r-1}{\underset{i=1}{\sum }}\overset{r}{\underset{j=i+1}{\sum }}h_{\left(i,j\right)}| \end{array}$$

where *r* = 21 is the number of elements of the variable *C*.

### Applying the analytical approaches to the biologic data

Artifacts and baseline drift in the ECG signal were removed and the R-wave peaks were detected. We divided the RR-interval time series into multiple epochs with length of 2.5 min and then computed periodicity for each epoch using our proposed measures. Also we divided the period when the rat was ventilated into four parts to test whether the quantified changes in HRV developed progressively during ventilation. The periodicity indices of the BVV- and CMV- treated rats during baseline, ventilation, and recovery periods were compared using repeated measures ANOVA test.

## Results

### Standard HRV measures of biologic data

**Figure 2:**
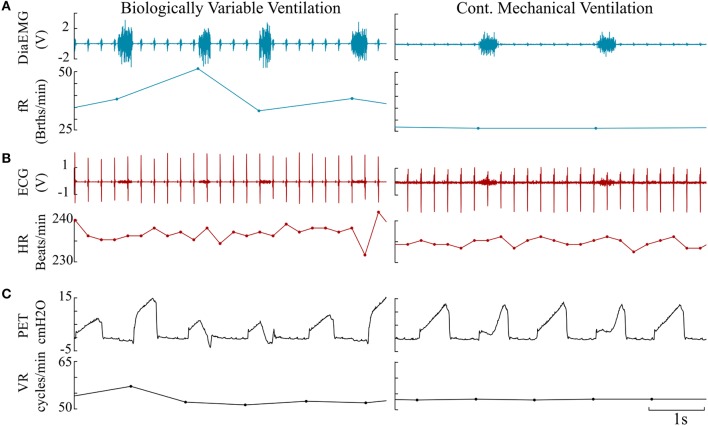
Representative paired traces consisting of raw data and derived instantaneous rates measured from BVV and CMV rats. **(A)** Diaphragm electromyogram (DiaEMG, Volt) paired with instantaneous respiratory frequency (fR, Breaths/minute). **(B)** Electrocardiogram (ECG) & heart rate (HR, Beats/minute). **(C)** Endotracheal pressure (PET, cm H2O) & ventilator rate (VR, Cycles/minute).

**Figure 3:**
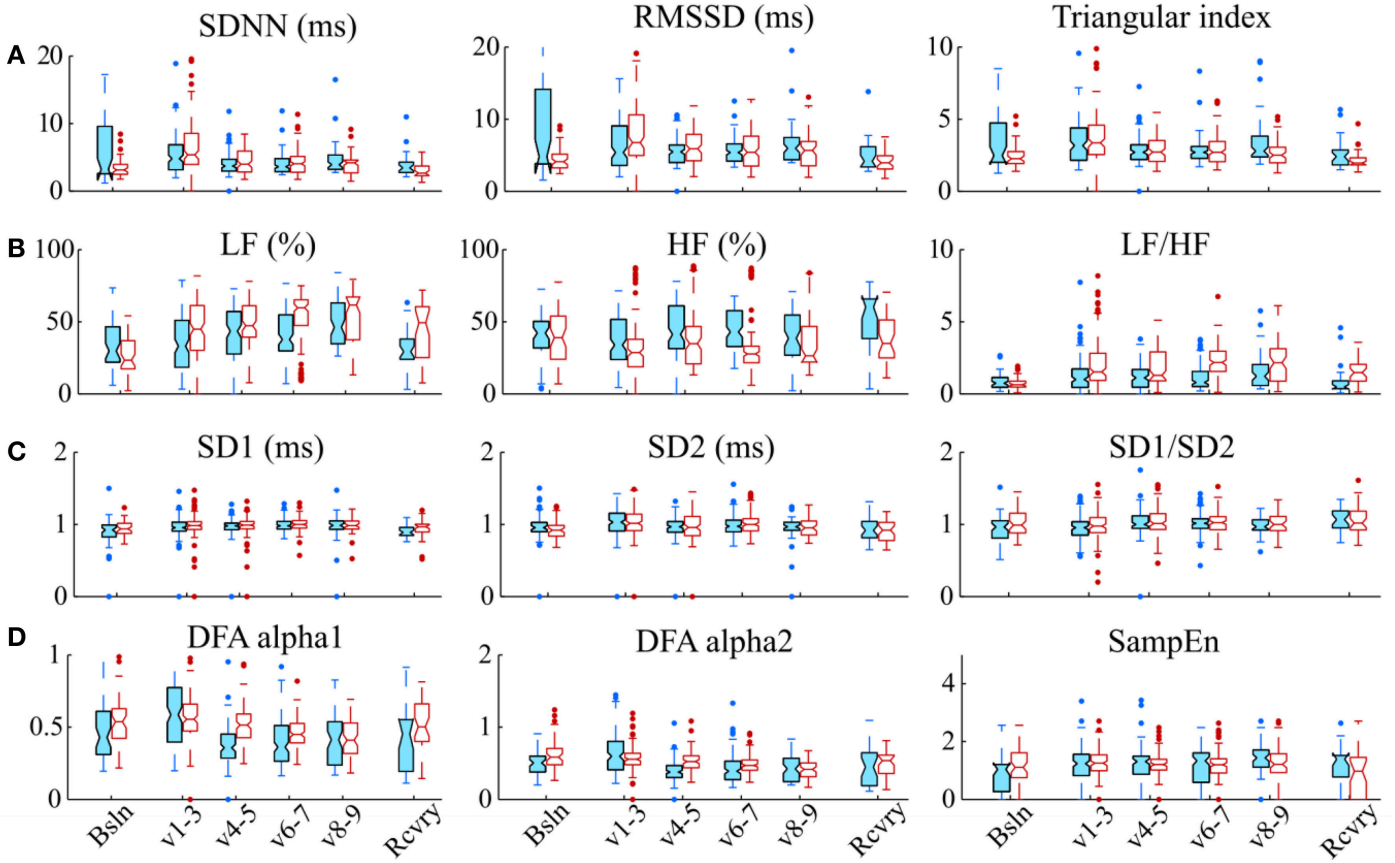
Various HRV measurements do not distinguish modes of ventilation. **(A)** Time domain methods: standard deviation of RR intervals **(**SDNN, ms), root mean square of the successive differences (RMSSD), and triangular index; **(B)** Frequency domain methods: relative power in low frequency band (LF, %); relative power in the high frequency band (HF, %), and the ratio of low-to-high-frequency power (LF/HF); **(C)** Analyses of Poincaré plots: the axis perpendicular to the line of identity (SD1), the axis on the line of identity SD2 and their ratio (SD1/SD2); and **(D)** Nonlinear methods: detrended fluctuation analysis (DFA), which has a short-term fractal exponent (alpha1) and a long-term fractal exponent (alpha2), and sample entropy (SampEn). These indices of HRV were measured during baseline (Bsln), ventilation (v1-3,1st h; V4-5, 2nd h; V6-7, 3rd h; & V8-9, 4th h) and recovery (Rcvry). These indices were not significantly different between BVV and CMV groups.

Representative signals recorded from the rats during BVV and CMV are presented in Figure 2. Time domain analyses of HRV are presented in Figure 3A. Figure 3B illustrates frequency domain analyses. Figures 3C,D present the results of Poincaré plot analysis and of nonlinear analyses respectively. These measures did not have significant differences for HRV between ventilator modes. In summary, the common time and frequency domain HRV measures did not differentiate HRV between lung injured rats treated by BVV and CMV.

### Synthetic data

**Figure 4:**
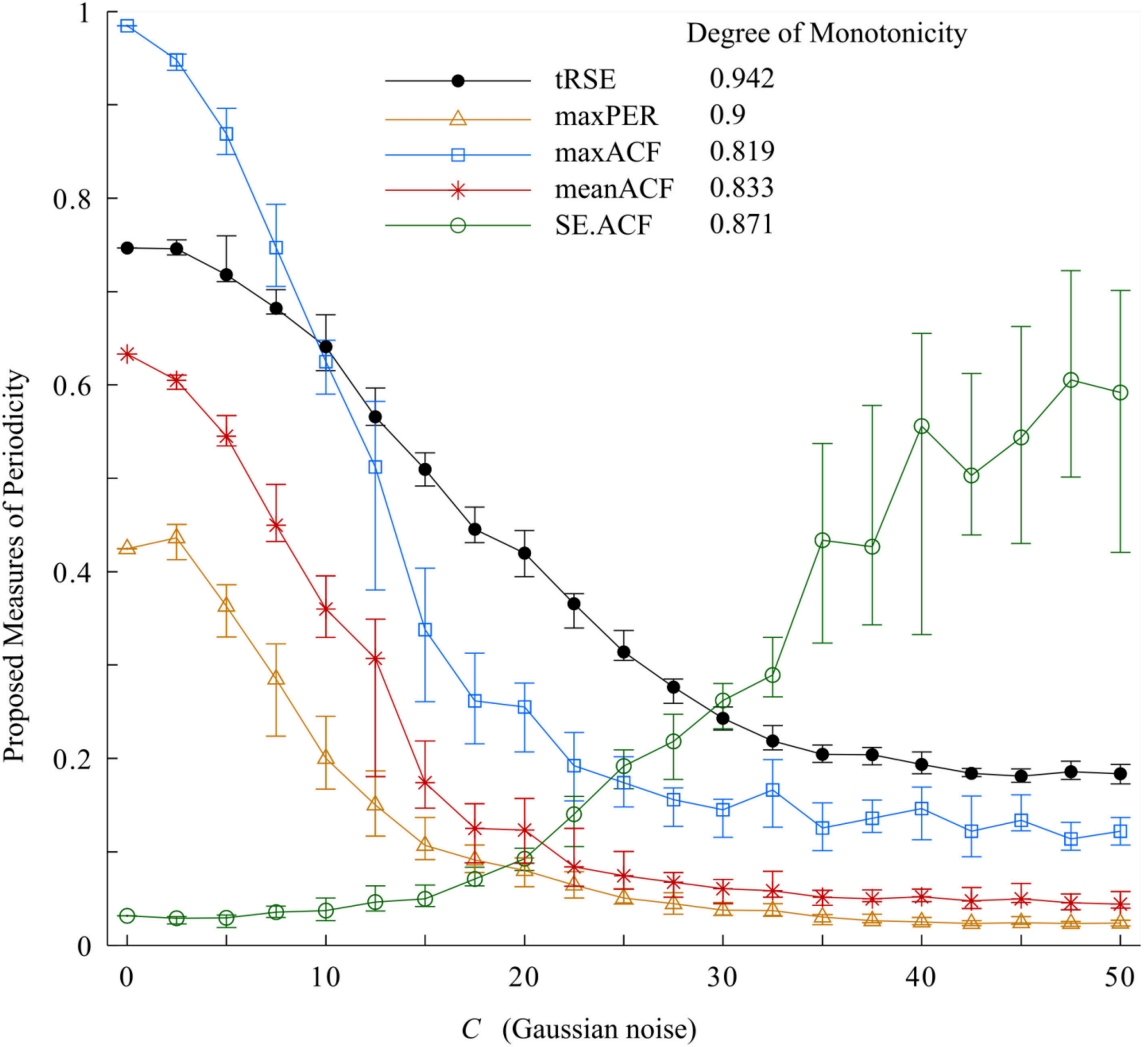
Variability indices and the degree of monotonicity. Of these measurements, tRSE was sensitive to monotonicity over a broad bandwidth.

The results of the variability measures and degree of monotonicity of the Van der Pol oscillator are presented in Figure 4. From this plot, as *C* increased, widening the bandwidth in the 0.5-1.5 Hz frequency range and increasing the variability; tRSE, maxPER, maxACF, and meanACF decreased and SE.ACF increased. The values of the indices depended on the range of *C* but each periodicity measure had a degree of monotonicity >0.7. The most sensitive in detecting changes in bandwidth was tRSE with a degree of monotonicity of 0.995. Further, tRSE decreased almost linearly over a wide range of values of *C* (5–37.5); whereas maxPER, maxACF, and meanACF had sigmoidal shapes decreasing rapidly over a narrow range of *C* (2.5–22.5). This behavior may be described by a Gaussian model. On the other hand, SE.ACF had a threshold (*C* = 17.5) and then increased linearly but with high variability if *C* > 32.5. Thus, tRSE performed well over a wide range of bandwidths, whereas maxPER, meanACF, and maxACF were limited to those cases with a narrow bandwidth; and SE.ACF was limited to those cases with a medium bandwidth.

### Periodicity in BVV and CMV treated ALI rats

**Figure 5:**
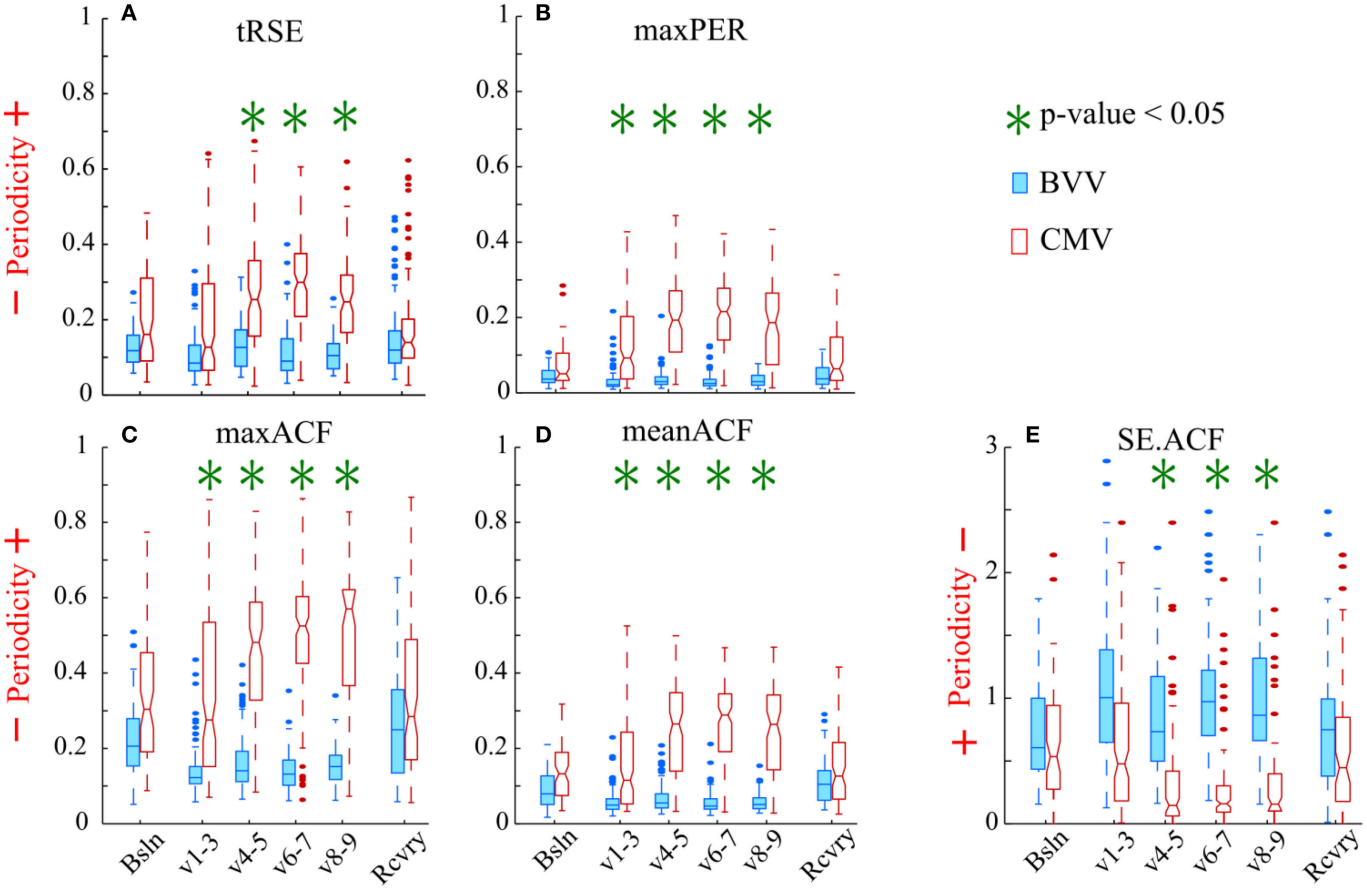
Variability of the RR intervals was greater during BVV than CMV by our measures of periodicity in the autocorrelograms. The box-and-whisker plots display the values for: **(A)** tRSE, **(B)** maxPer, **(C)** maxACF, **(D)** meanACF, and **(E)** Sample entropy of the acutocorrelation function (SE.ACF). **(A–D)** For these indices values closer to 1 indicate stronger periodicity. **(E)** Values of SE.ACF closer to zero have decreased probability in predicting the occurrence of the next heartbeat. We analyzed sequential RR intervals from baseline (Bsln), ventilation (V1-3, V4-5, V6-7, V8-9), and recovery (Rcvry) periods for BVV and CMV treated groups. HRV was significantly different during the two type of ventilation. HRV was not significant different during baseline and recovery.

All proposed periodicity measures consistently distinguished a difference in HRV during the two different ventilator protocols (Figure 5). The variables maxPER, maxACF, and meanACF were significantly greater during the 1st through 4th parts of the ventilatory period; whereas tRSE and SE.ACF were statistically different in only the last three parts of ventilation. The difference between the entropy measures versus simple measures of the peak and mean indicates that periodicity of HRV develops in the first hour of CMV, and then persists. All measures were similar during baseline and the recovery period. Thus, the decreased periodicity during BVV was associated with an enhanced high frequency component in the power spectral density of HRV during recovery. These data suggest that CMV has a strong influence on the periodicity of HRV that results in increases in the linear correlation of RR-intervals during ventilation, whereas BVV induces a more variable cardiac rhythm and that these effects on HRV were limited to the period of ventilation.

## Discussion

**Figure 6:**
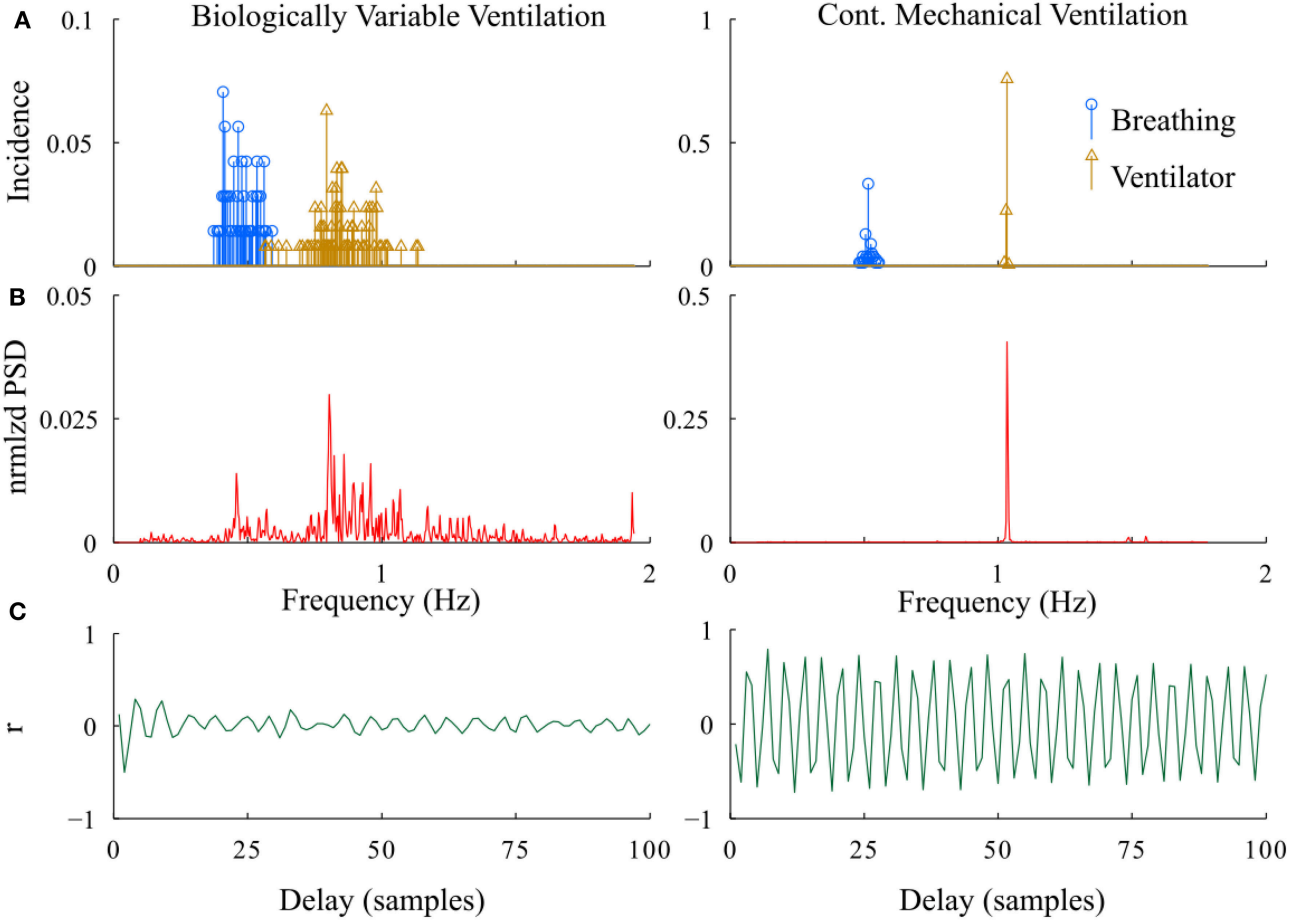
Representative examples of differences in the periodicity of heart rate during BVV and CMV. **(A)** Histograms of instantaneous fR (breathing) and VR (ventilator). **(B)** Power spectral density of the RR interval. **(C)** Autocorrelation plots of RR interval. As expected, the distribution of VR was much wider during BVV than CMV and this was reflected not only in the distribution of spontaneous fR but also in the PSD of the RR intervals, which were distinctly different. These differences were reflected in the autocorrelation plots which illustrated beat-to-beat variability during BVV but periodicity during CMV.

In developing the proposed methodologies, we compared the relative power spectrum of the RR- interval signals as well as the instantaneous frequency of ventilator and breathing patterns from the BVV and CMV groups following lung injury (Figure 6). The power spectrum of the RR-interval time series from a BVV treated rat (2.5 min segment) had a wide frequency range; whereas that from the CMV treated rat had a narrow bandwidth with a dominant peak at the ventilator frequency. Differences between BVV- and CMV- treated rats were also observed in the autocorrelation plots of the RR-interval time series. The autocorrelation plot for the BVV treated rat was flat; indicating that the signal was irregular while that of the CMV treated rat fluctuated at a regular interval, implying that the signal was periodic. In summary, the RR-interval time series of rats treated with non-varying ventilation (CMV group) oscillated with a relatively constant frequency whereas that of the BVV group treated with variable lung inflation was irregular. Thus, we concluded that the RR-interval time series of the BVV group had more variability than the CMV group.

**Figure 7:**
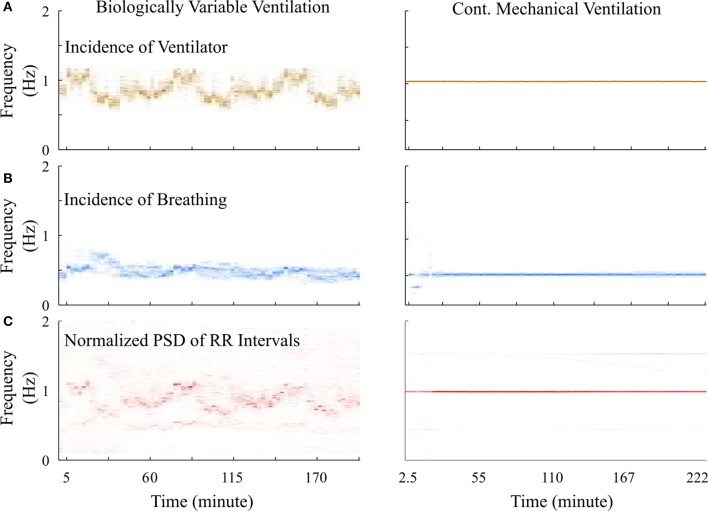
Time-frequency plot of signals measured from a representative BVV and CMV treated rats. **(A)** Frequency of ventilator were varied during BVV but fixed during CMV. **(B)** Respiratory frequency of CMV treated rat was constant and was half of the frequency of ventilator. However, the respiratory frequency of the BVV treated rat was relatively independent to the frequency of ventilator. **(C)** Frequency of RR interval signal of both BVV and CMV treated rat were coupled with the frequency of the ventilator rather than the respiratory frequency. The coupling caused the RR interval signal during BVV to be more non-stationary.

However, it was puzzling that this difference was not identified using standard HRV analyses. We observed the time-frequency plot of the ventilator, RR-interval, and breathing signals of BVV and CMV treated rats. The plots (Figure 7A) clearly show the varying and constant frequency of ventilator in the BVV and CMV mode, respectively. Interestingly, the frequency of the RR-interval signals (Figure 7B) was more tightly coupled with the frequency of the ventilator rather than the respiratory signal (Figure 7C) in both BVV- and CMV- treated rats. The coupling makes the RR-interval signal non-stationary; hence, the application of frequency domain measures of HRV with fixed frequency bands were not appropriate because the frequency of the RR-interval changed with the frequency of the ventilator.

Time and frequency domain HRV analyses do not necessarily distinguish between different types of variability in a time series, i.e., differences that result from the temporal dependence of a series of beats. Thus, these measures were not able to distinguish differences in HRV between the BVV and CMV rats. Biologic variability has a random or stochastic component but also a deterministic component, which is apparent in CMV as a repetitive pattern associated with the periodic ventilator rhythm. Thus, we developed analytical methods to quantify periodicity of the RR-interval time series, i.e., methods that would quantitate the deterministic variability in the CMV pattern that was evident in its periodogram and autocorrelation plot. In other words, the periodic heartbeats during CMV had a narrow support (bandwidth) around the ventilator frequency as well as a repetitive and regular autocorrelation function, while the heartbeat periodogram during BVV had wider support (bandwidth) and an autocorrelation function with a rapid decay and irregular pattern. Measures used to quantify the periodogram were tRSE and maxPER; differences in the autocorrelation function were quantified by maxACF, meanACF, and SE.ACF.

Before we applied our approach to measure periodicity of RR-interval time series in the physiologic data set, we tested them on simulated data using the Van der Pol oscillator with variable bandwidth. As the bandwidth of the signal increased, the degree of monotonicity decreased for tRSE, maxPER, maxACF, and meanACF (>0.7), while SE.ACF increased (degree of monotonicity ~0.8). Thus, these tools were sensitive to changes in the structure of the synthetic data in which variability in the form of noise was added to the bandwidth.

When these methods were applied to measure periodicity in the RR-interval time series recorded from rats after acute lung injury, they were able to identify differences in the variability of the RR-interval time series between the BVV and CMV groups; even though the conventional time and frequency domain HRV methods could not distinguish any differences between the groups. In both groups of rats, the generation of spontaneous breathing was coupled to mechanical ventilation. Most common was a spontaneous breath every other mechanical inflation (1:2 coupling), and during CMV the RR-interval time series had a strong periodicity at the ventilator frequency. In contrast during BVV, the relative power of the RR-interval time series was associated with both the frequencies for spontaneous breaths and mechanical inflation. Therefore, during CMV the periodicity of HRV was much more apparent at the ventilator rate as quantified by increasing tRSE, maxPER, maxACF, and meanACF; during BVV, the RR-interval time series had a broad range of low-frequencies as quantified by increasing SE.ACF during ventilation. Based on these analyses we concluded that BVV preserved more physiological-like variability in the RR-interval time series in lung-injured rats compared to during CMV.

Fetal monitoring of ECG provided the first example of the clinical relevance of conventional HRV (Hon and Lee, 1963b). Hon and Lee (1963a) showed that non-variable heartbeats were prognostic for fetal mortality. This finding was so dramatic that ultrasonic fetal monitors began to incorporate an autocorrelation function to enhance the signal-to-noise ratio of the fetal ECG from other bio-signals such a uterine contractions and noise in measuring fetal heart rate in external ultrasound recordings (Divon et al., 1985). But ironically in this case autocorrelation confounded HRV, and external recordings of fetal ECG were limited compared to direct recordings of the fetal ECG (Fukushima et al., 1985).

A reduction in HRV could lead to a false positive, i.e., incorrectly identifying a fetus at risk. We theorize that a quantitative analysis of the autocorrelogram as proposed in our approach may have distinguished false positives from true positives, especially in measuring the sustained periodicity of the autocorrelogram. While we hypothesized that HRV would be greater during BVV than CMV, this would be due to the strength of cardioventilator coupling during CMV. During BVV HRV increased because the ventilatory signal was variable. Cardioventilator coupling influenced HRV during both types of ventilation. However, while spontaneous breaths are also coupled to the ventilator, this manifested as 1 breath to 2 ventilator cycles and the magnitude of HRV at this lower respiratory frequency was weak. Our approach to measure periodicity reflects the entrainment of heart beats as well as spontaneous breaths with lung inflation.

In summary, we introduced a novel approach to quantify the variability in a heartbeat time series, beyond the conventional time and frequency domain HRV metrics. The proposed methods included the transform of Relative Shannon Entropy (tRSE), the maximum value of the RR-interval periodogram (maxPER), and the maximum and mean values, and sample entropy of the autocorrelation function (maxACF, meanACF, and SE.ACF, respectively). We evaluated these approaches using synthetic data generated by a Van der Pol oscillator with adjustable bandwidth. The results showed that tRSE, maxPER, maxACF, and meanACF monotonically decreased and SE.ACF increased as the bandwidth of the signal increased. We applied these novel methods to compare the effect of BVV and CMV on the variability of the RR-interval time series of rats after acute lung injury using both conventional HRV measures and the novel measure proposed in the paper. While we observed an effect of the type of ventilation on the RR-interval time series it was not detected by the established time and frequency domain HRV measures. Measuring periodicity of the RR-interval time series provided an opportunity to test our hypothesis that the BVV group had a higher RR-interval variability than the CMV group.

In analyzing the RR-interval variability, and even the variability of any time-series data, we recommend applying a series of complementary tools. Of course, certain tools have been suggested for specific applications; such as: power spectral density to evaluate autonomic tone, multiscale entropy to predict survival outcome in inflammatory shock (Vandendriessche et al., 2014, 2017), Poincaré plots to visualize variability in the distribution of successive time series points, and tRSE to evaluate periodicity as established in this work. However, a complete tool box to assess variability should contain methods to assess variability in both the time and frequency domains, as well as nonlinear measures to assess the complexity of the dynamics. We have described here analytical methods to assess periodicity in time series data, with applications to quantifying variability in RR-interval time series in rats with BVV and CMV.

## Data availability statement

The datasets and Matlab code for this study can be found in the github https://github.com/athungtong/Periodicity-A-Characteristic-of-HRV.

## Author contributions

All authors were involved in the conception and design of the study. MK contributed on all lab work. FJ and TD provided advice on lab work. AT contributed on all signal processing work. KL provided advice on signal processing. All authors were involved in the interpretation of the data. AT wrote the majority of the manuscript. TD and KL contributed to editing and approved the final version of the manuscript.

### Conflict of interest statement

The authors declare that the research was conducted in the absence of any commercial or financial relationships that could be construed as a potential conflict of interest.
